# Developmental and behavioural associations of burns and scalds in children: a prospective population-based study

**DOI:** 10.1136/archdischild-2016-311644

**Published:** 2016-11-13

**Authors:** Alan Emond, Clare Sheahan, Julie Mytton, Linda Hollén

**Affiliations:** 1 Scar Free Foundation Children's Burns Research Centre, University of Bristol, Bristol, UK; 2 Centre for Child and Adolescent Health, University of Bristol, Bristol, UK; 3 Centre for Child and Adolescent Health, University of the West of England, Bristol, UK

**Keywords:** burns, injury, child, development, behaviour, co-ordination, emotional regulation, Injury Prevention

## Abstract

**Objective:**

To investigate child developmental and behavioural characteristics and risk of burns and scalds.

**Design:**

Data on burns in children up to 11 years from 12 966 participants in the Avon Longitudinal Study of Parents and Children were linked to developmental profiles measured before the burn injury.

**Measures:**

Preinjury profiles of the children derived from maternal questionnaires completed in pregnancy, and at 6, 18, 42, 47 and 54 months. Injury data collected by questionnaire at 6, 15 and 24 months and 3.5, 4.5, 5.5, 6.5, 8.5 and 11 years of age.

**Results:**

Incidence: Burn rates were as follows: birth–2 years 71.9/1000/year; 2–4.5 years 42.2/1000/year; 5–11 years 14.3/1000/year. Boys <2 years were more likely to sustain burns, and girls had more burns between age 5 and 11 years. Medical attention was sought for 11% of burn injuries. *Development*: Up to age 2 years, burns were more likely in children with the most advanced gross motor developmental scores and the slowest fine motor development. Children with coordination problems at 4.5 years of age had increased risk of burns between 5 and 11 years. No associations were observed with cognitive skills. *Behaviour*: At 3.5 years, the Strengths and Difficulties Questionnaire scores and reported frequent temper tantrums predicted subsequent burns in primary school age. After adjustment for confounders, burns in the preschool period were related to gender and motor development, and in school-aged children, to frequent temper tantrums, hyperactivity and coordination difficulties.

**Conclusion:**

Child factors associated with increased risk of burns were male gender in infancy and female gender at school age, advanced gross motor development, coordination difficulties, hyperactivity and problems with emotional regulation.

**What is already known on this topic?**

- Burns and scalds are common injuries to young children, and the environmental and family risk factors for burn injury are well described.
- Few studies have investigated the developmental and behavioural characteristics of the child related to the risk of sustaining a burn.
- Most published studies of paediatric burn injury come from series of children who have attended emergency departments or been admitted to hospital.

**What this study adds?**

- Analysis of parental reports of 12 966 participants in a large representative population-based birth cohort showed peak burn incidence rates in the home of 72/1000 children/year, with only 11% being medically attended.
- The child's gender affected burn risk differently at different ages: boys <2 years were more likely to sustain burns, and girls had more burns between 5 and 11 years of age.
- Developmental and behavioural profiles of the children collected *before* the burn injury demonstrated that motor development, coordination difficulties, hyperactivity and problems with emotional regulation had consistent independent effects on burn injury risk.

## Background

The importance of burns and scalds as a cause of morbidity and mortality in young children is well documented.^1–3^ Every year over 50 000 children in the UK visit hospitals as a result of burns,^4^ and although many of these are minor injuries, 45% of all severe burns or scalds occur in children under 5 years of age. Most of the data regarding paediatric burn injury rates come from series of children who have attended emergency departments (EDs)^4^ or have been admitted to hospital.^5^ These data are biased by the health seeking behaviour of the parents, and social factors such as distance from home to the nearest ED, or whether the family have access to a car. Many burn incidents are entirely managed at home, but very few population studies have considered these more minor injuries.^6^

Factors related to paediatric injuries include individual characteristics such as younger age and male sex,^6–8^ family factors such as single parent and number of other children in the home^9^ ^10^ and social factors such as area deprivation.^11^ Few studies^12^ ^13^ have investigated the developmental and behavioural characteristics of the child related to the risk of sustaining a burn. This information is important to understand why children have such high rates of burn injury, and to highlight which children at which ages are particularly vulnerable, to inform preventative strategies.

The objectives of this study are to describe the incidence and distribution of burns sustained at home in children up to 11 years in a representative population cohort, and to define developmental and behavioural factors at different ages affecting the likelihood of sustaining a burn.

## Methods

### Sample

The Avon Longitudinal Study of Parents and Children (ALSPAC) is an ongoing population-based cohort study. Pregnant women resident in the former Avon Health Authority with an estimated date of delivery between 1 April 1991 and 31 December 1992 were invited to take part, resulting in a cohort of 14 062 live births and 13 988 children alive at 12 months.^14^ Ethical approval for the ALSPAC study was obtained from the local research ethics committees and this study was monitored by the ALSPAC Law and Ethics Committee. More detailed information on the ALSPAC study is available on the website: http://www.bristol.ac.uk/alspac, which contains details of all the data that are available through a fully searchable data dictionary.^15^

### Social and demographic background

Child and family features were prospectively collected before the burn injury. Maternal education and marital status were derived from questionnaires completed in pregnancy, and maternal parenting score (higher is better) was derived from questionnaires at 6 and 42 months. The Indices of Multiple Deprivation (IMD) were used based on the address of the family at different injury questionnaire time points: higher IMD scores indicate greater social deprivation. The ALSPAC Family Adversity Index (FAI, a derived index including 18 items reflecting family socioeconomic status) was used: the higher the FAI score, the greater the family adversity.

### Developmental measures

The child's developmental progress was measured by the ALSPAC Development Scale (a parental questionnaire derived from the Denver Developmental Screening Test^16^); we used the gross motor and fine motor scales at 6, 18 and 42 months. A variable for handedness, based on six questions, was derived from parental questionnaire at 42 months. Coordination problems were reported by parents in a questionnaire at 54 months. The child's IQ and selective attention were measured at 8 years in a research clinic, using a short version of the WISC III^17^ and the Tests of Everyday Attention for Children.^18^ The child's behavioural profile was measured using the Strengths and Difficulties Questionnaire (SDQ)^19^ completed by a parent at 47 months. We used the hyperactivity and conduct problems subscales, as externalising behaviours have been shown to be associated with increased risk of injury.^20^ Children were classified as having problems if categorised as ‘abnormal’ based on cut-offs for each SDQ subscale suggested by Goodman and Goodman.^21^ At 18 and 42 months, parents were also asked to rate how frequently their child had temper tantrums. The child's anxiety level at 7 years was reported by the parents using the Developmental and Well Being Assessment.^22^

### Outcome data

Burns were reported in parental questionnaires when the child was 6, 15 and 24 months, and 3.5, 4.5, 5.5, 6.5, 8.5 and 11 years of age. Parents were asked if their child had been burnt or scalded since the last questionnaire, and if so how many times. Further details were collected on the action taken by the person with the child (burn treated by themselves or sought medical attention).

### Statistical analyses

Analyses were conducted in STATA V.14 (StataCorp. 2015. *Stata Statistical Software: Release 14*. College Station, TX: StataCorp LP.) for three different age periods: infants aged 0–2 years, preschool children aged 2–4.5 years and primary school-aged children aged 5–11 years. Controls were those reported as having had no burn on all questionnaires within an age period.

Period prevalence, that is, the proportion of children sustaining a burn at some time during the specified age period (including multiple questionnaires), and incidence rates, defined as the number of new burns/person-time at risk, were calculated based on cases that responded to every questionnaire within each age period (complete follow-up). As follow-up time varied between questionnaires, we calculated person-time at risk separately for each questionnaire within an age period and then summed them to calculate the total. Prevalence rates are reported as percentages with 95% CIs. Incidence rates are reported as events/1000 children per year with 95% CI.

Using multivariable logistic regressions, we adjusted for confounders in a stepwise procedure: (1) adjusted for all child factors, (2) adjusted for all child factors and maternal factors, and (3) adjusted for child factors, maternal and household/socioeconomic factors. Gross motor and fine motor skills were entered as continuous predictors, and adjusted for age at questionnaire completion. For anxiety levels at age 7, IQ at age 8 and selective attention at age 8, we used a subset of burns between 9 and 11 to ensure that predictors were collected before the injury.

Multiple imputation (N=20 imputations) with chained equations using the ‘mi’ command was used to minimise bias due to missing data. Variables included in the imputation model were all of those included in the final logistic regression models, including the outcome, and those that predicted missingness in the predictors and confounders. We imputed our data up to the number of participants out of the total cohort that answered at least one of the burns questionnaires (N=12 996). More details are provided in online supplementary material S1.

10.1136/archdischild-2016-311644.supp1 Supplementary material

Analyses were performed using all available data, complete case dataset and multiple imputation. Regression results are presented using the imputed results only. Univariate and multivariable tables using all available data and complete case analyses are supplied in online supplementary tables S2 and S3.

## Results

### Sample

In total, 12 966 participants answered at least one of the burns questionnaires. The number of participants answering questions about burns varied from 11 433 at 6 months to 7415 at age 11 (see online supplementary table S4). As controls were those reported as having had no burn on all questionnaires within an age period, overall data were available for 9558 participants between birth and 2 years, 9039 between 2 and 4.5 years and 5906 between 5 and 11 years. Multiple burn injuries (>1 per age period) were reported for 8% (121/1484) of children with burns between birth and 2 years, 8% (75/992) between 2 and 4.5 years and 11% (86/795) between 5 and 11 years. The proportion of burn injuries in which medical attention was sought was 13.7% (189/1379) between birth and 2 years, 11.2% between 2 and 4.5 years, 7.7% (58/754) between 5 and 11 years.

### Missing data

Between 16% and 22% of participants had missing data on predictors and confounders between birth and 11 years, and 27%–55% were missing outcomes across the age groups (see online supplementary materials S1 and S5). The proportion with burns in the imputed dataset was slightly lower compared with that in the all available data analysis (14.2% vs 15.5%). Factors that strongly predicted missingness in the outcome were low maternal education, higher family adversity (FAI) and high socioeconomic deprivation (IMD). Other variables predicting missingness in predictors and confounders (all p<0.05) were young maternal age at delivery, high levels of antenatal depression, presence of domestic violence in pregnancy and non-white ethnicity.

### Prevalence

Period prevalence (table 1) between birth and 2 years was significantly higher than that between 2–4.5 and 5–11 years, particularly in boys. Overall prevalence between 2 and 4.5 years was no different to that at 5–11 years, but prevalence in girls aged 5–11 years was slightly higher than that in boys aged 5–11 years. No gender difference was seen between 2 and 4.5 years.

**Table 1:** Period prevalence and incidence rate of burns at the three different age periods, using cases with complete follow-up*

			Period prevalence % (95% CI)			Incidence rate (per 1000/year (95% CI))		
Period (months)	Population at risk	New cases	Total	Boys	Girls	Total	Boys	Girls
0–6	9339	142	13.5 (12.9 to 14.3)	15.2 (14.2 to 16.2)	11.8 (10.9 to 12.8)	71.9 (68.0 to 75.9)	81.1 (75.4 to 87.1)	62.1 (57.0 to 67.6)
6–15	9197	542						
15–24	8655	581						
24–38	8933	602	9.9 (9.3 to 10.6)	10.3 (9.4 to 11.2)	9.6 (8.7 to 10.5)	42.2 (39.5 to 45.1)	43.7 (39.9 to 47.8)	40.6 (36.8 to 44.7)
38–54	8331	284						
54–65	5658	132	9.7 (8.9 to 10.5)	8.8 (7.8 to 9.9)	10.6 (9.5 to 11.8)	14.3 (13.1 to 15.5)	12.9 (11.4 to 14.6)	15.7 (13.9 to 17.5)
65–77	5526	88						
77–103	5438	117						
103–140	5321	210						

*Complete follow-up refers to those participants responding to every questionnaire within each of the three age periods: 0–24 months=9339, 24–54 months=8933, 54–140 months=5658.

### Incidence

The rates of burns were highest between birth and 2 years with an overall incidence of 71.9/1000/year, dropping to 42.2 between 2 and 4.5 years, and to 14.3 between 5 and 11 years. Girls had lower rates than boys before 2 years, but slightly higher rates after 5 years (table 1).

### Maternal and socioeconomic factors

Online supplementary material S2 contains tables showing proportions and means of the maternal and socioeconomic factors included in our analyses. Multivariable analysis (table 2) showed that the maternal factors most consistently associated with increased risk of child burn injury in infancy and the preschool period were higher maternal education, never having been married and high family adversity as measured by the FAI. Better maternal parenting score at 6 m showed a protective effect on risk of burns between birth and 2 years, but not between 2 and 4.5 years. Between 5 and 11 years, the only confounder that showed any association with burns was maternal education, as mothers with a degree were more likely to report burns to their children. IMD scores were not associated with burn injury at any age.

**Table 2:** Multivariable results from imputed data set (N=12 996)

			Unadjusted		Adjusted 1*		Adjusted 2†		Adjusted 3‡		
Measure (non-reference group)	Age outcome (years)	Age at measure (months)	OR	95% CI	OR	95% CI	OR	95% CI	OR	95% CI	p Value
Gender (male)	0–2	Birth	1.34	1.19 to 1.50	1.32	1.18 to 1.48	1.33	1.18 to 1.49	1.32	1.18 to 1.49	<0.001
Gross motor score	0–2	6	1.03	1.02 to 1.04	1.03	1.02 to 1.04	1.03	1.02 to 1.04	1.03	1.02 to 1.03	<0.001
Fine motor score	0–2	18	0.98	0.96 to 1.00	0.98	0.96 to 0.99	0.98	0.96 to 1.00	0.99	0.97 to 1.01	0.13
Gender (male)	2–4.5	Birth	1.11	0.98 to 1.27	1.10	0.97 to 1.26	1.10	0.97 to 1.26	1.10	0.97 to 1.26	0.15
Gross motor score	2–4.5	6	1.01	1.00 to 1.02	1.01	1.00 to 1.02	1.01	1.00 to 1.02	1.01	1.00 to 1.02	0.11
Fine motor score	2–4.5	18	0.98	0.96 to 1.01	0.98	0.96 to 1.00	0.98	0.96 to 1.00	0.98	0.96 to 1.01	0.16
Temper tantrums (often)	2–4.5	18	1.26	0.99 to 1.61	1.26	0.99 to 1.60	1.27	0.99 to 1.61	1.16	0.91 to 1.48	0.23
Gender (male)	5–11	Birth	0.78	0.64 to 0.95	0.78	0.64 to 0.95	0.78	0.64 to 0.95	0.78	0.64 to 0.95	0.02
Gross motor score	5–11	42	0.98	0.95 to 1.01	0.98	0.95 to 1.00	0.98	0.95 to 1.01	0.98	0.95 to 1.01	0.11
Fine motor score	5–11	42	1.00	0.98 to 1.03	1.02	1.00 to 1.04	1.02	1.00 to 1.04	1.02	1.00 to 1.04	0.05
Temper tantrums (often)	5–11	42	1.60	1.20 to 2.14	1.43	1.06 to 1.93	1.44	1.07 to 1.95	1.41	1.04 to 1.92	0.03
Hyperactivity (abnormal)	5–11	47	1.36	1.11 to 1.67	1.25	1.01 to 1.54	1.26	1.02 to 1.55	1.24	1.01 to 1.54	0.04
Conduct problems (abnormal)	5–11	47	1.40	1.16 to 1.69	1.24	1.01 to 1.54	1.23	0.99 to 1.53	1.20	0.96 to 1.50	0.10
Coordination problems (yes)	5–11	54	1.86	1.31 to 2.62	1.70	1.22 to 2.37	1.71	1.23 to 2.38	1.69	1.21 to 2.35	0.002
Handedness (left/mixed)	5–11	42	0.95	0.78 to 1.16	0.93	0.75 to 1.15	0.94	0.76 to 1.16	0.93	0.76 to 1.15	0.51

Full model 0–2 years, OR (95% CI) for confounders; maternal education (degree): 1.34 (1.14 to 1.58), maternal parenting score (worst decile): 1.19 (1.00 to 1.41), marital status (never married): 1.38 (1.20 to 1.60), FAI: 1.11 (1.07 to 1.15), IMD (worst quintile): 1.09 (0.91 to 1.31).

Full model 2–4.5 years, OR (95% CI) for confounders; maternal education (degree): 1.35 (1.12 to 1.62), maternal parenting score (worst decile): 0.89 (0.69 to 1.15), marital status (never married): 1.45 (1.22 to 1.72), FAI: 1.06 (1.02 to 1.10), IMD (worst quintile): 1.08 (0.86 to 1.35).

Full model 5–11 years, OR (95% CI) for confounders: maternal education (degree): 1.36 (1.12 to 1.65), maternal parenting score (worst decile): 1.26 (0.99 to 1.60), marital status (never married): 1.06 (0.83 to 1.34), FAI: 1.05 (1.00 to 1.11), IMD (worst quintile): 0.98 (0.73 to 1.30).

Motor scores are entered as continuous variables.

*Adjusted 1: all child factors.

†Adjusted 2: adjusted 1 + maternal education, maternal parenting score.

‡Adjusted 3: adjusted 2 + marital status, FAI and IMD. p Values reported for adjustment 3 only.

FAI, Family Adversity Index; IMD, Index of Multiple Deprivation.

### Child factors

The results of multivariable analyses using the imputed dataset of child factors associated with burn injury at the different ages are shown in table 2. Results were similar using all available data and the complete case dataset (see online supplementary tables S3 and S6).

*Gender*: Boys were more likely to sustain a burn between birth and 2 years, whereas there was no difference between boys and girls in the age period of 2–4.5 years. Girls were slightly more likely to sustain burns in the school age period.

**Figure 1:**
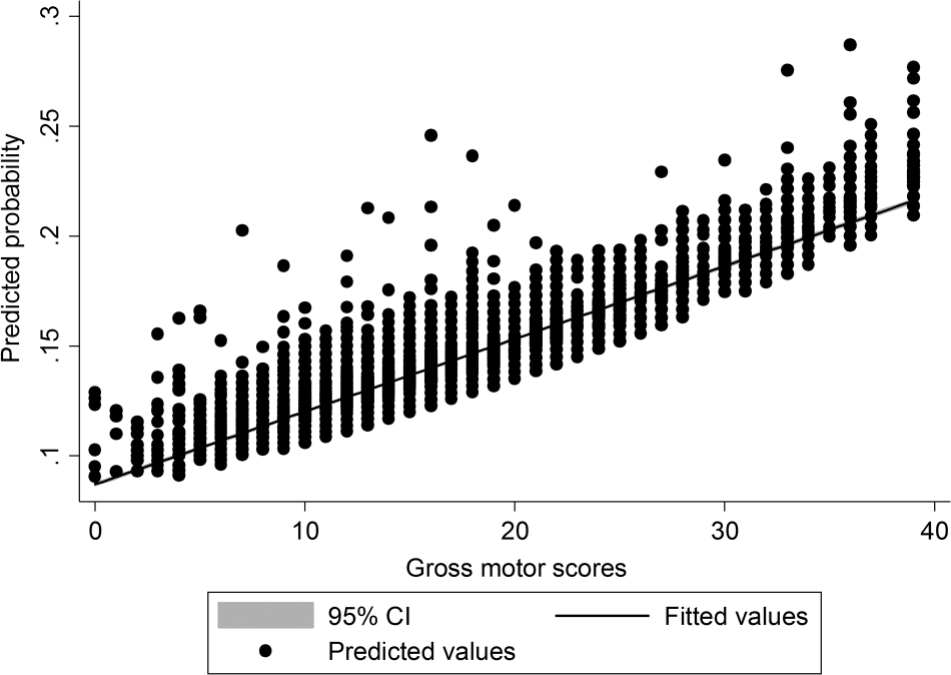
Probability predictions of gross motor scores affecting risk of burns between birth and 2 years.

**Figure 2:**
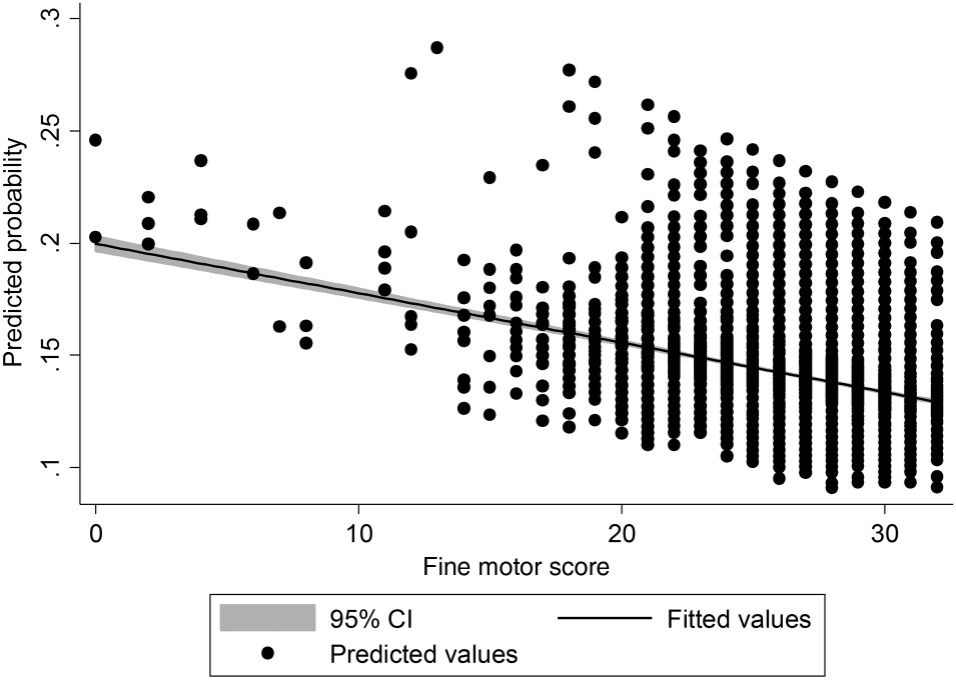
Probability predictions of fine motor scores affecting risk of burns between birth and 2 years.

*Motor development*: Children with the most advanced gross motor developmental scores reported at 6 months were more likely to sustain burns between birth and 2 years (table 2 and figure 1). The odds of a burn increased by 3% for every one-point increase in motor scores. Fine motor development showed the opposite pattern, with the most advanced fine motor development being associated with a decrease in odds of a burn, but this effect was weaker and only apparent when adjusting for gross motor scores (figure 2). The effect of gender and gross motor scores remained after adjustment for confounders, whereas adjustment for family and socioeconomic factors attenuated the association with fine motor scores. Children with reported coordination problems at 4.5 years were much more likely to sustain burns between ages 5 and 11. Left-handed children did *not* have any increased risk of a burn between 5 and 11 years.

*Cognitive development*: No associations were observed between total IQ scores (OR (95% CI): 1.01 (1.00 to 1.01)) and attention scores at 8 years (1.12 (0.79 to 1.59)) with burns in the subset of children aged 9–11 years.

*Emotional regulation*: Frequent temper tantrums at 18 months were not associated with burns in the preschool period, but reported tantrums at 42 months predicted an increased risk of burns between ages 5 and 11. Maternal report of general child anxiety symptoms at age 7 showed no association with increased risk of burns at ages 9–11 (OR: 0.74 (0.33 to 1.69)).

*Behavioural profile*: The SDQ completed by the parent at 47 months was predictive of subsequent burns: hyperactivity scores showed consistent associations with injury between ages 5 and 11. Conduct difficulties were also associated with increased risk of burns, but this association attenuated after adjustment.

## Discussion

This prospective study is unique because it has captured burn injuries in the home environment, with information on the child's development and behaviour collected before the injury occurred. Burns to children aged below 11 years in the home environment were commonly reported, with a peak in the age period of 15–24 months. The child's gender affected burns risk differently at different ages, and motor development, coordination difficulties and problems with hyperactivity and emotional regulation had consistent independent effects on burn injury risk.

The strengths of this study are that it uses a large representative population-based birth cohort, with burns prospectively captured by parental report, thereby avoiding biases associated with attending hospital. The developmental and behavioural characteristics of the children were collected before the burn injury, and a range of family and social covariates were available for adjustment. There are few population-based studies of burns in children,^23–25^ most are based on hospital attendance,^4^ and none have prospectively collected information on development and behavioural profile before the injury.

Limitations are that the parental reports were not validated, and the recall period was variable for different questionnaires. Since parents are more likely to recall severe events than minor ones with the elapse of time,^26^ recall bias could make the results for minor burns less accurate, and mothers who were better educated reported more injuries. Overall, the study is likely to have underestimated the true incidence of paediatric burns. There were limited data on the severity of the burn, so that seeking medical attention was used as a proxy for injury severity. No information was available on whether the burns were intentional or related to neglect or maltreatment.^27^ ^28^ The most important limitation is missing data due to loss to follow-up, which is inevitable in a study like this, so multiple imputation was used to correct for any biases introduced by attrition.

The peak incidence in burns in the second year of life is well documented, but the continuing injury rates reported through school age were higher than would be predicted from ED attendances.^4^ The gender differences changed noticeably with increasing age, and could be a result of girls being more exposed to thermal hazards in the kitchen than boys.^5^ The associations of burn injury with motor development may not surprise clinicians, but there is little literature on the connection between motor development and burns. The peak incidence of burns around 15 months, particularly in those with advanced gross motor skills, may reflect that this is the age when exploratory behaviour is greatest.^29^ In contrast, poor coordination (perceived clumsiness) was related to burns risk in the older children. Hertog *et al*^7^ attributed clumsiness as being contributory in 57% of scald injuries in their cohort of children aged 0–4 years, although individual differences in motor ability were not analysed. Activity levels and emotional regulation were the most important behavioural factors affecting burn risk at later ages. We showed consistent effects of hyperactivity and to a lesser extent conduct difficulties on burn risk during school age, consistent with studies showing that activity levels in children with attention deficit hyperactivity disorder are related to higher injury levels,^20^ although not all burn studies have shown an effect of hyperactivity.^30^

## Conclusions and implications

Minor burns are common in young children, and over 80% do not seek medical attention, illustrating the need for promoting good first aid practices in the home.^31^ Reducing hazards by environmental modification and limiting exposure by improving supervision are crucial strategies to prevent thermal injuries, but individual child characteristics are also independent risk factors, and change through childhood. As the child gets older, individual characteristics become relatively more influential than family or environmental factors. Anticipatory guidance is essential in educating parents about changing burn risks associated with developmental progress,^32^ and clinicians need to highlight to parents the increased risk of burns in those children with difficulties in motor coordination, emotional regulation and behavioural control.
