# Supplementary material for: Developmental and behavioural associations of burns and scalds in children: a prospective population-based study
Source: Arch Dis Child. 2016 Nov 13;102(5):428–83. doi: 10.1136/archdischild-2016-311644 (PMC6234232; doi:10.1136/archdischild-2016-311644)
Supplement: Supplementary material [file archdischild-2016-311644supp001.pdf]

Supplementary material 1. Missing data and multiple imputation

Missing data is a problem in longitudinal cohort studies such as ALSPAC. 35.3% of the original live births had missing data on burn outcome between birth and 2, 39% between 2 and 4.5 years, and 60% between 5 and 11. Considering the number of cases who reported at least one burn outcome between birth and age 11 (N=12996), the corresponding figures were 26.5%, 30.5% and 54.6%.

Of those children with complete outcome data (burn yes/no), 84 % of children between birth and 2 years had no missing values in any of the predictors or confounders, 14% were missing on 1 and 2% were missing on 2 or more. Between 2-4.5 years, 78% had no missing values, 14% missing on 1 and 8% missing on 2 or more predictors or confounders. Between 5 and 11, the numbers were 83%, 12% and 5% respectively. The degree of missingness varied between variables (see tables S2 below).

Those with missing outcome data differed on a number of predictors and other potential confounders to those with outcome data (table S5). Hence, it is likely that without taking this into account, our results would be biased. Multiple imputation is a common technique used to correct for bias introduced by loss to follow-up (Sterne et al. 2009). We used multiple imputation by chained equations using the "mi" command in Stata v.14 (StataCorp. 2015). We imputed up to the number of participants who had reported at least one burn between birth and age 11 (N = 12996) using 20 imputations. Imputation model diagnostics were performed using the command 'midiagplots' which compares the distributions of the observed, imputed, and completed values (Eddings & Marchenko 2012). Binary variables and categorical variables used logistic, and ordinal regression, as appropriate, specified in the mi impute command. Normally distributed variables were incorporated using linear regression, and other continuous skewed variables were included using predictive mean matching (N=5 set as the number of closest observations/nearest neighbours from which to draw imputed values) in the imputation model. To identify auxiliary variables, we used logistic regressions to explore if the following factors were associated with missingness in our predictors and confounders: maternal age at delivery, levels of postnatal depression (8 weeks), presence of domestic violence in pregnancy, maternal education level, family adversity (as measured by the ALSPAC Family Adversity Index), socioeconomic deprivation (as measured by the Index of Multiple Deprivation) and ethnicity. All these variables were highly associated with missingness ($p<0.05$), helping to support the assumption of "missing at random", and were included in the final imputation model, together with those included in the final logistic

regression models. We also included all burn outcome variables up to age 11 as well as earlier and later measures of the predictor variables.

References

Sterne JA, White IR, Carlin JB, Spratt M, Royston P, Kenward MG, Wood AM, Carpenter JR. Multiple imputation for missing data in epidemiological and clinical research: potential and pitfalls. *BMJ* 2009; 38 :b2393.

Eddings W, Marchenko Y. Diagnostics for multiple imputation in Stata. *Stata Journal* 2012; 12(3):353

Supplementary tables S2: UNIVARIABLE analyses using different datasets.

Table S.2.1 Pre-injury child, family and socio-economic characteristics of the children sustaining a burn between birth and 2 years compared to controls. Categorical variables are reported as percentages [95% confidence intervals] and continuous variables as mean [standard error]. IMD = Index of Multiple Deprivation.

**Variables**	**Age at measure**	**All available data**		**Complete case**		**Imputed**	
		Control (N_{max}=8074)	Burn (N_{max}=1484)	Control (N=6856)	Burn (N=1160)	Control (N=11156)	Burn (N=1840)
**% Outcome**		84.5	15.5	85.5	14.5	85.8	14.2
**Child factors**							
Gender - Male	*birth*	50.7 [49.6, 51.8] *N=8074*	58.1 [55.6, 60.6] *N=1484*	50.8 [49.7, 52.0]	58.7 [55.8, 61.5]	50.5 [49.6, 51.5]	57.7 [55.2, 60.3]
Gross motor score	*6m*	16.1 [0.06] *N =8041*	17.1 [0.17] *N=1398*	16.0 [0.07]	17.0 [0.16]	16.3 [0.06]	17.3 [0.17]
Fine motor score	*18m*	26.7 [0.04] *N=7885*	26.6 [0.08] *N=1401*	26.7 [0.04]	26.6 [0.08]	26.7 [0.03]	26.5 [0.09]
**Covariates**							
Maternal education - Degree	*32w gest*	14.5 [13.8, 15.3] *N=7903*	16.2 [14.4, 18.2] *N=1411*	14.7 [13.9, 15.5]	17.2 [15.1, 19.4]	12.8 [12.1, 13.4]	14.9 [13.2, 16.6]
Maternal parenting score - Worst decile	*6m*	10.4 [9.8, 11.1] *N=8059*	12.7 [11.0, 14.5] *N=1404*	10.1 [9.4, 10.9]	12.2 [10.5, 14.3]	11.3 [10.6, 11.9]	13.4 [11.7, 15.2]
Marital status - Never married	*8w gest*	13.5 [12.8, 14.3] *N=7937*	22.5 [20.4, 24.7] *N=1445*	13.4 [12.6, 14.3]	20.3 [18.0, 22.7]	17.1 [16.4, 17.9]	25.1 [22.8, 27.4]
Family Adversity Index	*8-32w gest*	1.1 [0.02] *N=8074*	1.5 [0.05] *N=1484*	1.1 [0.02]	1.4 [0.05]	1.2 [0.02]	1.6 [0.05]
IMD (quintiles) - Most deprived	*6m*	17.7 [16.8, 18.6] *N=7296*	22.5 [20.3, 24.9] *N=1270*	17.0 [16.1, 17.9]	21.6 [19.3, 24.0]	20.8 [19.9, 21.6]	25.7 [23.4, 28.0]

Table S.2.2. Pre-injury child, family and socio-economic characteristics of the children sustaining a burn between 2 and 4.5 years compared to controls.

Categorical variables are reported as percentages [95% confidence intervals] and continuous variables as mean [standard error]. IMD = Index of Multiple Deprivation.

Variables	Age at measure	All available data		Complete case		Imputed	
		Control (N_{max}=8047)	Burn (N_{max}=992)	Control (N=6688)	Burn (N=784)	Control (N=11672)	Burn (N=1324)
**% Outcome**		89.0	11.0	89.5	10.5	89.8	10.2
**Child factors**							
Gender<br>- Male	birth	51.4 [50.3, 52.5]	54.0 [50.9, 57.1]	51.2 [50.0, 52.4]	53.3 [49.8, 56.8]	51.3 [50.4, 52.2]	53.9 [50.9, 57.0]
Gross motor score	6m	16.1 [0.06]<br>*N=7700*	16.6 [0.19]<br>*N=933*	16.0 [0.07]	16.3 [0.20]	16.4 [0.06]	16.8 [0.20]
Fine motor score	18m	26.7 [0.03]<br>*N=7755*	26.6 [0.10]<br>*N=936*	26.7 [0.04]	26.6 [0.11]	26.7 [0.03]	26.5 [0.11]
Temper tantrums<br>- Often	18m	27.6 [26.7, 28.7]<br>*N=7704*	29.3 [26.4, 32.3]<br>*N=931*	27.6 [26.6, 28.7]	28.9 [25.8, 32.1]	28.9 [28.0, 29.8]	31.0 [27.6, 34.4]
**Covariates**							
Maternal education<br>- Degree	32w gest	14.7 [13.9, 15.5]<br>*N=7830*	16.8 [14.6, 19.3]<br>*N=964*	15.0 [14.2, 15.9]	18.4 [15.8, 21.3]	12.8 [12.2, 13.5]	15.3 [13.2, 17.5]
Maternal parenting score<br>- Worst decile	6m	10.5 [9.8, 11.2]<br>*N=7719*	9.4 [7.7, 11.4]<br>*N=936*	10.3 [9.6, 11.1]	9.3 [7.5, 11.6]	11.6 [11.0, 12.3]	10.9 [8.7, 13.1]
Marital status<br>- Never married	8w gest	14.1 [13.4, 14.9]<br>*N=7894*	21.5 [19.0, 24.2]<br>*N=962*	13.4 [12.6, 14.3]	20.1 [17.4, 23.0]	17.5 [16.8, 18.2]	24.9 [22.1, 27.6]
Family Adversity Index	8-32w gest	1.1 [0.02]<br>*N=8047*	1.2 [0.05]<br>*N=992*	1.1 [0.02]	1.3 [0.06]	1.2 [0.01]	1.5 [0.05]
IMD (quintiles)<br>- Most deprived	38m	18.4 [17.5, 19.3]<br>*N=7463*	19.6 [17.1, 22.4]<br>*N=892*	17.1 [16.2, 18.0]	18.3 [15.7, 21.1]	21.6 [20.8, 22.5]	22.4 [19.8, 25.0]

Table S.2.3. Pre-injury child, family and socio-economic characteristics of the children sustaining a burn between 5 and 11 years compared to controls.

Categorical variables are reported as percentages [95% confidence intervals] and continuous variables as mean [standard error]. IMD = Index of Multiple Deprivation.

**Variables**	**Age at measure**	**All available data**		**Complete case**		**Imputed**	
		% No (N_{max}=5111)	% Yes (N_{max}=795)	% No (N=4185)	% Yes (N=567)	% No (N=11668)	% Yes (N=1328)
**% Outcome**		86.5	13.5	88.1	11.9	89.8	10.2
**Predictors**							
Gender - Male	*birth*	51.0 [49.6, 52.4] *N=5111*	45.3 [41.8, 48.8] *N=795*	51.1 [49.6, 52.7]	47.1 [43.0, 51.2]	52.2 [51.2, 53.2]	46.1 [41.7, 50.6]
Gross motor score	*42m*	26.3 [0.05] *N=4981*	26.1 [0.13] *N=732*	26.3 [0.05]	26.0 [0.15]	26.4 [0.04]	26.1 [0.19]
Fine motor score	*42m*	29.9 [0.06] *N=4978*	30.0 [0.15] *N=732*	29.9 [0.06]	30.0 [0.17]	29.7 [0.04]	29.7 [0.18]
Temper tantrums - Often	*42m*	14.4 [13.5, 15.4] *N=4941*	18.8 [16.1, 21.8] *N=729*	14.1 [13.1, 15.2]	17.8 [14.9, 21.2]	16.3 [15.4, 17.1]	20.4 [17.4, 23.4]
Hyperactivity - Abnormal	*47m*	12.6 [11.7, 13.6] *N=4938*	17.3 [14.7, 20.2] *N=718*	12.6 [11.6, 13.7]	16.0 [13.2, 19.3]	14.3 [13.6, 15.1]	18.6 [15.9, 21.4]
Conduct problems - Abnormal	*47m*	11.6 [10.7, 12.5] *N=4938*	15.5 [13.0, 18.3] *N=718*	11.2 [12.3, 14.3]	14.6 [12.0, 17.8]	13.4 [12.7, 14.2]	17.8 [15.3, 20.4]
Coordination problems - Yes	*54m*	3.1 [2.6, 3.6] *N=4960*	5.4 [4.0, 7.3] *N=723*	3.0 [2.5, 3.6]	5.6 [4.0, 7.9]	3.6 [3.1, 4.0]	6.5 [4.6, 8.4]
Handedness - Left/mixed	*42m*	32.7 [31.4, 34.0] *N=4959*	32.1 [28.9, 35.6] *N=731*	32.7 [31.3, 34.1]	30.3 [26.7, 34.3]	35.7 [34.6, 36.7]	34.5 [30.2, 38.8]
**Covariates**							
Maternal education - Degree	*32w gest*	18.1 [17.1, 19.2] *N=5037*	19.7 [17.0, 22.7] *N=753*	18.3 [17.1, 19.5]	21.0 [17.8, 24.5]	12.8 [12.1, 13.4]	15.9 [13.6, 18.2]
Maternal parenting score - Worst decile	*18m*	8.4 [7.6, 9.2] *N=5013*	11.4 [9.3, 13.9] *N=747*	8.2 [7.4, 9.1]	9.7 [7.5, 12.4]	9.9 [9.2, 10.5]	12.5 [10.1, 14.8]
Marital status - Never married	*8w gest*	11.7 [10.8, 12.6] *N=5044*	15.2 [12.8, 17.9] *N=765*	11.3 [10.4, 12.3]	14.6 [12.0, 17.8]	18.0 [17.3, 18.8]	20.0 [16.7, 23.3]
Family Adversity Index	*8-32w gest*	0.97 [0.02] *N=5111*	1.23 [1.12, 1.34] *N=773*	0.94 [0.02]	1.15 [0.06]	1.25 [0.02]	1.43 [0.06]
IMD (quintiles) - Most deprived	*38m*	15.6 [14.6, 16.7] *N=4734*	18.7 [15.8, 21.9] *N=637*	14.8 [13.7, 15.9]	17.1 [14.2, 20.4]	22.4 [21.5, 23.3]	23.3 [19.8, 26.9]

Supplementary tables S3. MULTIVARIABLE results from analyses using different datasets.

Table S3.1. All available data analyses, reporting odds ratios (OR) and 95% confidence intervals (CI).

Measure (non-reference group)	Age outcome	Age at measure	Unadjusted		Adjusted 1*		Adjusted 2**		Adjusted 3***	
			**OR**	**95% CI**	**OR**	**95% CI**	**OR**	**95% CI**	**OR**	**95% CI**
Gender (male)	0-2y	Birth	1.35	1.20, 1.51	1.32	1.17, 1.48	1.32	1.17, 1.49	1.37	1.21, 1.56
Gross motor score	0-2y	6m	1.03	1.02, 1.04	1.03	1.02, 1.04	1.03	1.02, 1.04	1.03	1.02, 1.04
Fine motor score	0-2y	18m	0.98	0.97, 1.00	0.97	0.96, 0.99	0.98	0.96, 1.00	0.99	0.96, 1.01
Gender (male)	2-4.5y	Birth	1.11	0.97, 1.27	1.09	0.94, 1.25	1.09	0.95, 1.25	1.08	0.93, 1.25
Gross motor score	2-4.5y	6m	1.02	1.00, 1.03	1.01	1.00, 1.03	1.02	1.00, 1.03	1.02	1.00, 1.03
Fine motor score	2-4.5y	18m	0.99	0.97, 1.01	0.98	0.96, 1.01	0.98	0.96, 1.00	0.98	0.96, 1.00
Temper tantrums (often)	2-4.5y	18m	1.25	0.98, 1.60	1.23	0.96, 1.58	1.24	0.96, 1.59	1.24	0.96, 1.59
Gender (male)	5-11y	Birth	0.79	0.68, 0.92	0.81	0.69, 0.96	0.83	0.69, 0.98	0.87	0.72, 1.05
Gross motor score	5-11y	42m	0.98	0.96, 1.01	0.98	0.95, 1.00	0.98	0.95, 1.00	0.97	0.94, 1.00
Fine motor score	5-11y	42m	1.01	0.99, 1.03	1.02	0.99, 1.05	1.02	1.00, 1.05	1.03	1.00, 1.05
Temper tantrums (often)	5-11y	18m	1.30	1.14, 1.49	1.48	1.10, 1.99	1.48	1.10, 2.00	1.44	1.04, 1.99
Hyperactivity (abnormal)	5-11y	47m	1.83	1.54, 2.19	1.29	1.01, 1.63	1.28	1.01, 1.64	1.19	0.91, 1.55
Conduct problems (abnormal)	5-11y	47m	1.81	1.51, 2.17	1.25	0.97, 1.60	1.21	0.94, 1.56	1.17	0.89, 1.54
Coordination problems (yes)	5-11y	54m	1.80	1.26, 2.59	1.75	1.19, 2.57	1.79	1.22, 2.63	1.71	1.13, 2.58
Handedness (left/mixed)	5-11y	42m	0.97	0.82, 1.15	0.94	0.79, 1.13	0.95	0.80, 1.14	0.87	0.72, 1.06

* Adjusted 1: All child factors; **Adjusted 2: Adjusted 1 + maternal education, maternal parenting score; ***Adjusted 3: Adjusted 2 + marital status, FAI (Family Adversity Index) and IMD (Index of Multiple Deprivation).

OR [95% CI] for confounders full model 0-2 years; maternal education (degree): 1.30 [1.09, 1.55], maternal parenting score (worst decile): 1.23 [1.01, 1.50], marital status (never married): 1.40 [1.18, 1.66], FAI: 1.11 [1.07, 1.16], IMD (worst quintile): 1.10 [0.90, 1.34].

OR [95% CI] for confounders full model 2-4.5 years; maternal education (degree): 1.34 [1.10, 1.64], maternal parenting score (worst decile): 0.89 [0.69, 1.15], marital status (never married): 1.53 [1.26, 1.87], FAI: 1.06 [1.00, 1.11], IMD (worst quartile): 0.98 [0.76, 1.25].

OR [95% CI] for confounders full model 5-11 years: maternal education (degree): 1.22 [0.98, 1.52], maternal parenting score (worst decile): 1.11 [0.82, 1.50], marital status (never married): 1.19 [0.92, 1.54], FAI: 1.09 [1.02, 1.16], IMD (worst quartile): 1.13 [0.85, 1.50].

Gross motor and fine motor scores entered as continuous variables.

Table S3.2. **Complete case analyses, reporting odds ratios (OR) and 95% confidence intervals (CI).**

Measure (non-reference group)	Age outcome	Age at measure	Unadjusted		Adjusted 1*		Adjusted 2*		Adjusted 3*	
			**OR**	**95% CI**	**OR**	**95% CI**	**OR**	**95% CI**	**OR**	**95% CI**
Gender (male)	0-2y	Birth	1.37	1.21, 1.56	1.36	1.20, 1.54	1.36	1.20, 1.55	1.37	1.21, 1.56
Gross motor score	0-2y	6m	1.03	1.02, 1.04	1.03	1.02, 1.05	1.04	1.02, 1.05	1.03	1.02, 1.04
Fine motor score	0-2y	18m	0.99	0.97, 1.01	0.98	0.96, 1.00	0.98	0.96, 1.00	0.99	0.96, 1.01
Gender (male)	2-4.5y	Birth	1.08	0.93, 1.26	1.07	0.93, 1.25	1.08	0.93, 1.25	1.08	0.93, 1.25
Gross motor score	2-4.5y	6m	1.01	1.00, 1.02	1.01	1.00, 1.03	1.01	1.00, 1.03	1.01	0.99, 1.02
Fine motor score	2-4.5y	18m	0.98	0.96, 1.01	0.98	0.95, 1.00	0.98	0.95, 1.00	0.98	0.95, 1.00
Temper tantrums (often)	2-4.5y	18m	1.17	0.90, 1.52	1.17	0.90, 1.52	1.18	0.90, 1.53	1.11	0.85, 1.44
Gender (male)	5-11y	Birth	0.85	0.71, 1.01	0.86	0.72, 1.03	0.86	0.71, 1.03	0.87	0.72, 1.05
Gross motor score	5-11y	42m	0.98	0.95, 1.00	0.97	0.94, 1.00	0.97	0.95, 1.00	0.97	0.94, 1.00
Fine motor score	5-11y	42m	1.01	0.99, 1.03	1.02	1.00, 1.05	1.02	1.00, 1.05	1.03	1.00, 1.05
Temper tantrums (often)	5-11y	42m	1.61	1.18, 2.19	1.49	1.08, 2.05	1.49	1.08, 2.06	1.44	1.04, 1.99
Hyperactivity (abnormal)	5-11y	47m	1.32	1.04, 1.69	1.20	0.92, 1.56	1.21	0.93, 1.57	1.19	0.91, 1.55
Conduct problems (abnormal)	5-11y	47m	1.36	1.06, 1.75	1.22	0.93, 1.60	1.22	0.93, 1.60	1.17	0.89, 1.54
Coordination problems (yes)	5-11y	54m	1.93	1.29, 2.87	1.79	1.19, 2.70	1.78	1.18, 2.68	1.71	1.13, 2.58
Handedness (left/mixed)	5-11y	42m	0.90	0.74, 1.09	0.88	0.72, 1.07	0.89	0.73, 1.08	0.87	0.72, 1.06

[*] Adjusted 1: All child factors; [**]Adjusted 2: Adjusted 1 + maternal education, maternal parenting score; [***]Adjusted 3: Adjusted 2 + marital status, FAI (Family Adversity Index) and IMD (Index of Multiple Deprivation).

OR [95% CI] for confounders full model 0-2 years; maternal education (degree): 1.30 [1.09, 1.55], maternal parenting score (worst decile): 1.23 [1.01, 1.50], marital status (never married): 1.40 [1.18, 1.66], FAI: 1.11 [1.07, 1.16], IMD (worst quintile): 1.10 [0.90, 1.34].

OR [95% CI] for confounders full model 2-4.5 years; maternal education (degree): 1.34 [1.10, 1.64], maternal parenting score (worst decile): 0.89 [0.69, 1.15], marital status (never married): 1.53 [1.26, 1.87], FAI: 1.06 [1.00, 1.11], IMD (worst quartile): 0.98 [0.76, 1.25].

OR [95% CI] for confounders full model 5-11 years: maternal education (degree): 1.22 [0.98, 1.52], maternal parenting score (worst decile): 1.11 [0.82, 1.50], marital status (never married): 1.19 [0.92, 1.54], FAI: 1.09 [1.02, 1.16], IMD (worst quartile): 1.13 [0.85, 1.50].

Gross motor and fine motor scores entered as continuous variables.

Supplementary table S4. Period prevalence of burns at each questionnaire time point.

Period (months)	N answering questionnaire	Period prevalence N (%)*	Period prevalence – complete follow up**
**0-6**	11433	172 (1.5)	142 (1.5)
**6-15**	11014	683 (6.2)	568 (6.1)
**15-24**	10360	784 (7.6)	695 (7.4)
**24-38**	10066	676 (6.7)	602 (6.7)
**38-54**	9681	424 (4.4)	392 (4.4)
**54-65**	8980	191 (2.1)	132 (2.3)
**65-77**	8496	160 (1.9)	95 (1.7)
**77-103**	8264	190 (2.3)	134 (2.4)
**103-140**	7415	336 (4.5)	246 (4.3)

* Proportion of participants reporting a burn/total number answering the burns questionnaire within each period.

** Proportion of participants reporting a burn/total number that responded to every questionnaire within each of the three age periods (complete follow-up): 0-24 months = 9339, 24-54 months = 8933, 54-140 months = 5658.

Supplementary table S5. **Distribution of predictors and confounders included in the final models between those who reported burn outcomes and those that did not.**

Sample size varies depending on completeness of each variable. Continuous variables are reported using means and standard error and categorical variables as proportions with 95% confidence intervals.

**Variables**	**With outcome**	**N (max 9558)**	**Missing outcome**	**N (max 3438)**	**P-value***
0-2y					
Gender<br>- Male	51.9 [50.8, 52.9]	9558	50.7 [49.0, 52.4]	3438	0.25
Gross motor score	16.2 [0.06]	9439	17.4 [0.16]	1951	<0.001
Fine motor score	26.7 [0.08]	9286	26.7 [0.03]	1753	0.38
Maternal education<br>- Degree	14.8 [14.1, 15.5]	9314	7.6 [6.6, 8.7]	2496	<0.001
Maternal parenting score<br>- Worst decile	10.8 [10.2, 11.4]	9463	14.3 [12.9, 15.9]	1978	<0.001
Marital status<br>- Never married	14.9 [14.2, 15.6]	9382	28.6 [26.9, 30.3]	2705	<0.001
Family Adversity Index	1.16 [0.02]	9558	1.60 [0.03]	3028	<0.001
Index of Multiple Deprivation<br>- Most deprived quintile	18.4 [17.6, 19.2]	8566	27.6 [25.5, 29.7]	1764	<0.001
	**With outcome**	**N (max 9039)**	**Missing outcome**	**N (max 3957)**	**P-value***
2-4.5y					
Gender<br>- Male	51.7 [50.7, 52.7]	9039	51.2 [49.6, 52.7]	3957	0.58
Gross motor score	16.1 [0.06]	8633	17.3 [0.13]	2757	<0.001
Fine motor score	26.7 [0.03]	8691	26.6 [0.07]	2348	0.39
Temper tantrums<br>- Often	27.8 [26.9, 28.8]	8635	30.9 [29.0, 32.8]	2329	0.01
Maternal education					

- Degree	14.9 [14.2, 15.7]	8794	8.4 [7.5, 9.4]	3016	<0.001
Maternal parenting score<br>- Worst decile	10.4 [9.8, 11.0]	8655	14.5 [13.2, 15.9]	2786	<0.001
Marital status<br>- Never married	15.0 [14.2, 15.7]	8856	26.3 [24.8, 27.8]	3231	<0.001
Family Adversity Index	1.14 [0.02]	9039	1.59 [0.03]	3547	<0.001
Index of Multiple Deprivation<br>- Most deprived quintile	18.5 [17.7, 19.4]	8355	27.4 [24.7, 30.2]	1045	<0.001

	**With outcome**	**N (max 5906)**	**Missing outcome**	**N (max 7090)**	**P-value***
5-11y					
Gender<br>- Male	50.2 [49.0, 51.5]	5906	52.6 [51.5, 53.8]	7090	0.006
Gross motor score	26.3 [0.05]	5713	26.4 [0.06]	4293	0.04
Fine motor score	29.9 [0.05]	5710	29.5 [0.06]	4289	<0.001
Temper tantrums<br>- Often	15.0 [14.1, 15.9]	5670	17.7 [16.6, 18.9]	4272	<0.001
Hyperactivity<br>- Abnormal	13.2 [12.3, 14.1]	5656	15.4 [14.3, 16.6]	3856	0.002
Conduct problems<br>- Abnormal	12.1[14.3, 16.6]	5656	14.5 [13.5, 15.7]	3856	<0.001
Coordination problems<br>- Yes	3.3 [2.9, 3.9]	5683	4.0 [3.5, 4.7]	3977	0.09
Handedness<br>- Left/mixed	32.7 [31.4, 33.9]	5690	37.7 [36.2, 39.1]	4260	<0.001
Maternal education<br>- Degree	18.3 [17.4, 19.4]	5790	8.4 [7.7, 9.1]	6020	<0.001
Maternal parenting score<br>- Worst decile	8.8 [8.1, 9.5]	5760	11.1 [10.2, 11.9]	5276	<0.001
Marital status<br>- Never married	12.2 [11.3, 13.0]	5809	23.4 [22.3, 24.4]	6278	<0.001
Family Adversity Index	1.00 [0.02]	5884	1.50 [0.02]	6702	<0.001
Index of Multiple Deprivation<br>- Most deprived quintile	16.0 [15.0, 17.0]	5371	24.8 [23.2, 26.5]	2536	<0.001

* P-values are reported using Chi-square tests for categorical variables and t-tests for continuous variables.

Supplementary table S6. Comparison of results from the fully adjusted model 3 between complete case analysis and multiple imputation analysis.

Measure (non-reference group)	Age outcome	Complete case		Multiple Imputation	
		**OR**	**95% CI**	**OR**	**95% CI**
Gender (male)	0-2y	1.37	1.21, 1.56	1.32	1.18, 1.49
Gross motor score	0-2y	1.03	1.02, 1.04	1.03	1.02, 1.03
Fine motor score	0-2y	0.99	0.96, 1.01	0.99	0.97, 1.01
Gender (male)	2-4.5y	1.08	0.93, 1.25	1.10	0.97, 1.26
Gross motor score	2-4.5y	1.01	1.00, 1.02	1.01	0.99, 1.02
Fine motor score	2-4.5y	0.98	0.95, 1.00	0.98	0.96, 1.01
Temper tantrums (often)	2-4.5y	1.11	0.85, 1.44	1.16	0.91, 1.48
Gender (male)	5-11y	0.87	0.72, 1.05	0.78	0.64, 0.95
Gross motor score	5-11y	0.97	0.94, 1.00	0.98	0.95, 1.01
Fine motor score	5-11y	1.03	1.00, 1.05	1.02	1.00, 1.04
Temper tantrums (often)	5-11y	1.44	1.04, 1.99	1.41	1.04, 1.92
Hyperactivity (abnormal)	5-11y	1.19	0.91, 1.55	1.24	1.01, 1.54
Conduct problems (abnormal)	5-11y	1.17	0.89, 1.54	1.20	0.96, 1.50
Coordination problems (yes)	5-11y	1.71	1.13, 2.58	1.69	1.21, 2.35
Handedness (left/mixed)	5-11y	0.87	0.72, 1.06	0.93	0.76, 1.15

Gross motor and fine motor scores entered as continuous variables.
